# MiRNA-194 Regulates Palmitic Acid-Induced Toll-Like Receptor 4 Inflammatory Responses in THP-1 Cells

**DOI:** 10.3390/nu7053483

**Published:** 2015-05-13

**Authors:** Huiqun Tian, Chaoqi Liu, Xiaohua Zou, Wei Wu, Changcheng Zhang, Ding Yuan

**Affiliations:** 1 Hubei Key Laboratory of Tumor Microenvironment and Immunotherapy, China Three Gorges University, Yichang 443002, China; E-Mails: 756972975@qq.com (H.T.); ctgulcq@163.com (C.L.); 806030825@qq.com (X.Z.); lezhiweiwei@163.com (W.W.); 2 Medical College, China Three Gorges University, Yichang 443002, China

**Keywords:** palmitic acid, TRAF6, miR-194, TLR4

## Abstract

There is strong evidence to suggest that inflammatory responses link obesity and diseases, and the understanding of obesity-induced inflammatory mechanisms is central to the pathogenesis of diseases such asnonalcoholic fatty liver disease(NAFLD) and atherosclerosis that are modified by obesity. Based on this, anti-inflammatory treatments become a potential therapies for obesity-related diseases like NAFLD.A critical role of toll-like receptor (TLR) and its downstream molecules such as tumor necrosis factor receptor-associated factor 6(TRAF6) has been documented in inflammatory response induced by fatty acid. TLR pathway regulation provides a new insight to controlling the inflammatory response induced by fatty acid. Taken together, our study was aimed to understand the mechanism of fatty acid-mediated inflammation and look for an effective target which can prevent the inflammatory response induced by obesity. In this study, we used the saturated fatty acid palmitic acid (PA) to activate TLR4 signal pathway in human monocyte cells THP-1 that established an intracellular inflammatory model. Followed with activated TLR4, downstream molecular TRAF6 was upregulated and ultimately induced proinflammatory cytokine production. Based on this model, we also found that PA downregulated miR-194 expression with TLR4 activation. Moreover, our results showed that key signal molecular TRAF6 is a target of miR-194, overexpression of miR-194 directly decreased TRAF6 expression and attenuated the release of proinflammatory cytokine TNF-α in PA-activated monocyte THP-1. We conclude that miR-194 negatively regulates the TLR4 signal pathway which is activated by PA through directly negative TRAF6 expression.

## 1. Introduction

Followed by increased consumption of foods rich in saturated fatty acid and reduced physical activity, obesity-related diseases such as nonalcoholic fatty liver disease (NAFLD) and atherosclerosis have reached epidemic proportions around the world. Moreover, inflammation is well known to play a crucial role in the initiation and development of fat-related diseases [1]. For instance, expression of TNF-α, which is a common pro-inflammation cytokine, is upregulated in the pancreatic islets of patients and animal models with obesity [2,3]. Accumulation of macrophages within islets has been observed in NAFLD, atherosclerosis and obesity subjects [4].

The potential molecular between free fatty acids and obesity-related diseases is the toll-like receptors (TLRs). As major pattern recognition receptors (PRRs), TLRs comprise a highly conserved family of receptors that recognize pathogen-associated molecular patterns (PAMPs) and damage-associated molecular patterns (DAMPs) and trigger the immunity responses, which are abundantly located in macrophages [5,6]. Recent studies indicated that TLR4 signaling is important for fatty acid-mediated inflammatory response [7]. For instance, saturated fatty acids activating the inflammation via TLR4 in macrophage had been identified by Lee *et al.* and Suganami and Ogawa, respectively [8,9]. Saturated fatty acids serve as a natural endogenous ligand of TLR4, thereby activating downstream signal molecules, such as TRAF6, to mediated proinflammatory transcription factor nuclear factor-kappaB (NF-κB) activation and pro-inflammatory cytokines such as TNF-α production [10].

The proinflammation cytokines result in inflammatory responses that play a crucial role in the development of obesity-related diseases including NAFLD. Cytokines such as TNF-α and TGF-β from Kupffer cells (KCs) are required for the development of nonalcoholic steatohepatitis (NASH) [11]. With the methionine choline-deficient (MCD) diet, the TLR4 mutant mice showed decreased steatohepatitis compared with wild-type mice [12]. All of these data suggest that blocking intracellular TLR4 signaling represents a potential novel strategy for breaking the progression of NAFLD, as well as inflammation induced by the saturated fatty acid which is involved in many diseases.

MicroRNAs (miRs) are small, noncoding RNAs, 19–24 nucleotides in length, which posttranscriptionally regulate gene expression by targeting the 3′untranslated region (3′-UTR) of target mRNAs [13]. MiRs always prevent protein synthesis through degrading mRNAs and inhibit their translation [14]. Results of previous studies have identified a critical role for miRs in a TLR4 pathway such as miR-146 negatively regulating the pathway through targeting TRAF6 and IRAK1 [15]. Moreover, abnormal expression of miRs was observed in obesity-related diseases. These data suggest that miRs have a potential role in the control of the TLR4 pathway which is activated by fatty acid.

Palmitic acid (PA) is a saturated fatty acid. As one of the most highly abundant free fatty acids (FFA) in the modern diet, PA in the blood and in the white adipose tissue acts as a natural dietary ligand for activation of the TLR4 signal pathway, which ultimately leads to NF-κB activation in macrophage and promotes proinflammatory cytokine release [10].

The present study was undertaken to elucidate the potential involvement of miRs in an activated TLR4 pathway induced by PA. The results reveal that PA-induced TLR4 activating can be negatively regulated by miR-194 which targets TRAF6, a crucial molecular in the TLR4 pathway. Therefore, miR-194 can be developed as a potential target for intervention of inflammation response caused by free fatty acids.

## 2. Materials and Methods

### 2.1. Materials

Anti-TRAF6 and anti-TLR4 were purchased from Santa Cruz Biotechnology (United States). Palmitic acid and LPS were obtained from Sigma pharmaceuticals (Hamburg, Germany). The 1M palmitate stock solution was prepared in 0.1 mM NaOH by heating at 70 °C. A 10% FFA-free BSA (Sigma, St. Louis, MO, USA) solution was prepared in ddH_2_O and maintained at 55 °C in a water bath. Subsequently, 10 mM PA in 1% BSA solution was obtained by complexing the appropriate amount of palmitate stock solution to 10% BSA at 55 °C for another 30 min. The above solution was then cooled and filter sterilized, then stored at −20 °C until use.

### 2.2. Cell Culture

Human THP-1 cells were purchased from China Center for Type Culture Collection (Wuhan, China) and maintained in a humidified 5% CO_2_ atmosphere in RPMI1640 (Gibco (Invitrogen), Carlsbad, CA, USA) containing 10% natural bovine calf serum (NBCS) (Gibco (Invitrogen), Carlsbad, CA, USA), 100 U/mL penicillin and 100 μg/mL streptomycin. Cells were seeded in 12-well plates at a density of 2×10^5^ cells per well and incubated for 8 h in cell culture media containing certain concentration of palmitic acid. Control cells were incubated with the same medium containing the amount of solvent (BSA) used to dissolve the palmitic acid.

### 2.3. NF-κBLuciferase Reporter Analysis

NF-κB luciferase plasmid was obtained from our lab. NF-κB luciferase reporter plasmid was transfected to THP-1using Lipofectamine 2000 (Invitrogen) according to the manufacturer’s instructions. A full 24 h later, the cells were exposed to a different concentration of PA for different amount of time. Lyates from varyinggroups were mixed with substrate at a ratio of 1:10. The luciferase activity was measured using the Luciferase Assay System (Promega, Madison, WI, USA).The data are obtained from three independent experiments and presented as the fold increase in luciferase activities (means ± S.D.) relative to the control.

### 2.4. Analysis of Gene Expression

Total RNA for both mRNAs and miRs analysis was extracted from cells which exposed to 250 μM palmitic acid for 8 h using the Trizol reagent (Sigma) and reverse-transcribed into complementary DNA. For mRNA analyses, the first strand of cDNA was synthesized using random primers. For miRs RT-PCR, total RNA was converted into cDNA using specific primers (miR-194, 5′-GTCGTATCCAGTGCAGGGTCCGAGGTATTCGCACTGGATACGACTTCCACA-3′). Quantification of the complementary DNA template was performed with real-time PCR using SYBR green (Takara Bio, Dalian, China) as a fluorophore and semi-quantitative interpretation respectively (primers showed in Table 1). The semi-quantitative interpretation product was separated by electrophoresis at 100 V for 40 min through a 2% agarose gel and detected by ethidium bromide staining, finally quantified by KODAKMI software system. The result of quantitative real-time PCR was calculated using the 2^−ΔΔCT^ method.

**Table 1:** Lists of primers for PCR reaction.

Gene	Sequence of Primer (5′ to 3′)
GAPDH	Forward, ACTTCAACAGCGACACCCACTC
	Reverse, TCTCTCTTCCTCTTGTGCTCTTGC
TLR4	Forward, GGTGATTGTTGTGGTGTCCCA
	Reverse, AGTGTTCCTGCTGAGAAGGCG
TRAF6	Forward, GCTTTCCAGCGACCCACA
	Reverse, CCCTCCGAAGGCTACCCAT
TNF-α	Forward, CCCTCAGCAAGGACAGCAGA
	Reverse, AGCCGTGGGTCAGTATGTGAGA
TGF-β	Forward, GCAAGTGGACATCAACGGG
	Reverse, CGCACGCAGCAGTTCTTCT
miR-194	Forward, GCCCGCTGTAACAGCAACTCCAT
	Reverse, GTGCAGGGTCCGAGGT

### 2.5. Mature miRNA Transfections

THP-1 cells were cultured in a 12-well culture plate at a density of 2×10^5^ cells per well for 24 h, the cells were then transfected with 20 nM miR-194 mimic, miR-194 inhibitor and negative controls (GenaPharma, Shanghai, China) using Lipofectamine 2000 (Invitrogen, Carlsbad, CA, USA) according to the manufacturer’s instructions for 48 h.

### 2.6. Western Blot Analysis

After stimulation with 250 μM Palmitic acid, cells were lysed in 1%Nonidet P-40 lysis buffer (50 mM HEPES, pH 7.4, 150 mM NaCl, 2 mM EDTA, 1%Nonidet P-40, and protease inhibitors). The cell lysates were resolved by SDS-PAGE and transferred onto PVDF membranes. Membranes were blocked for 2 h with 5% skim milk in Tris-buffered saline containing 0.1% Tween 20 and incubated overnight at 4 °C with primary antibodies (the antibodies were diluted at a ratio of 1:1000). Membranes were washed, incubated for 45 min with appropriate secondary antibodies conjugated to horseradish peroxidase (the antibodies were diluted at a ratio of 1:3000), and developed using chemiluminescence substrates, finally detected by chemiluminescence (ECL). Protein expression was quantified using a KODAKMI software system.

### 2.7. ELISA Analysis

Supernatants of plasmid-transfected or PA-stimulated cells were harvested at the indicated time points. Human TGF-β and TNF-α in the supernatants (100 μL per sample) were quantified by a sandwich ELISA performed according to the manufacturer’s instructions (BD OptEIA ELISA set; BD Biosciences Pharmingen, Heidelberg, Germany).

### 2.8. Plasmid Construction and Luciferase Reporter Assays

For the 3′UTR luciferase reporter assay, the pGL3-CM Luciferase miRs Target Expression Vector (a gift from The Wang Laboratory, Carolina Cardiovascular Biology Center, Department of Cell and Dev) was used. The oligonucleotides were ligated into the MulI-XhoI site of pGL3-CM. Cells (3 × 10^5^ cells per well) were plated onto a six-well plate one day prior to transfection. The THP-1 cells were co-transfected with the miR-194 mimics/inhibitor (or their controls) and constructed pGL3-CM vectors using Lipofectamine 2000 (Invitrogen) according to the manufacturer’s instructions. After 24 h, the cells were exposed to PA for 8 h before harvest. The whole-cell extracts were then prepared for the luciferase assay. The luciferase activity was measured using the Luciferase Assay System (Promega, Madison, WI, USA).The data are obtained from three independent experiments and presented as the fold increase in luciferase activities (means ± S.D.) relative to the control.

### 2.9. Statistical Analysis

Data are presented as means ± SDs from at least three independent experiments. *p*-Values were determined using one-way analysis of variance followed by the Tukey-Kramer test. *p* < 0.05 were considered statistically significant.

## 3. Results

### 3.1. PA-Activated TLR4 Signal Pathway in THP-1 Cells

**Figure 1:**
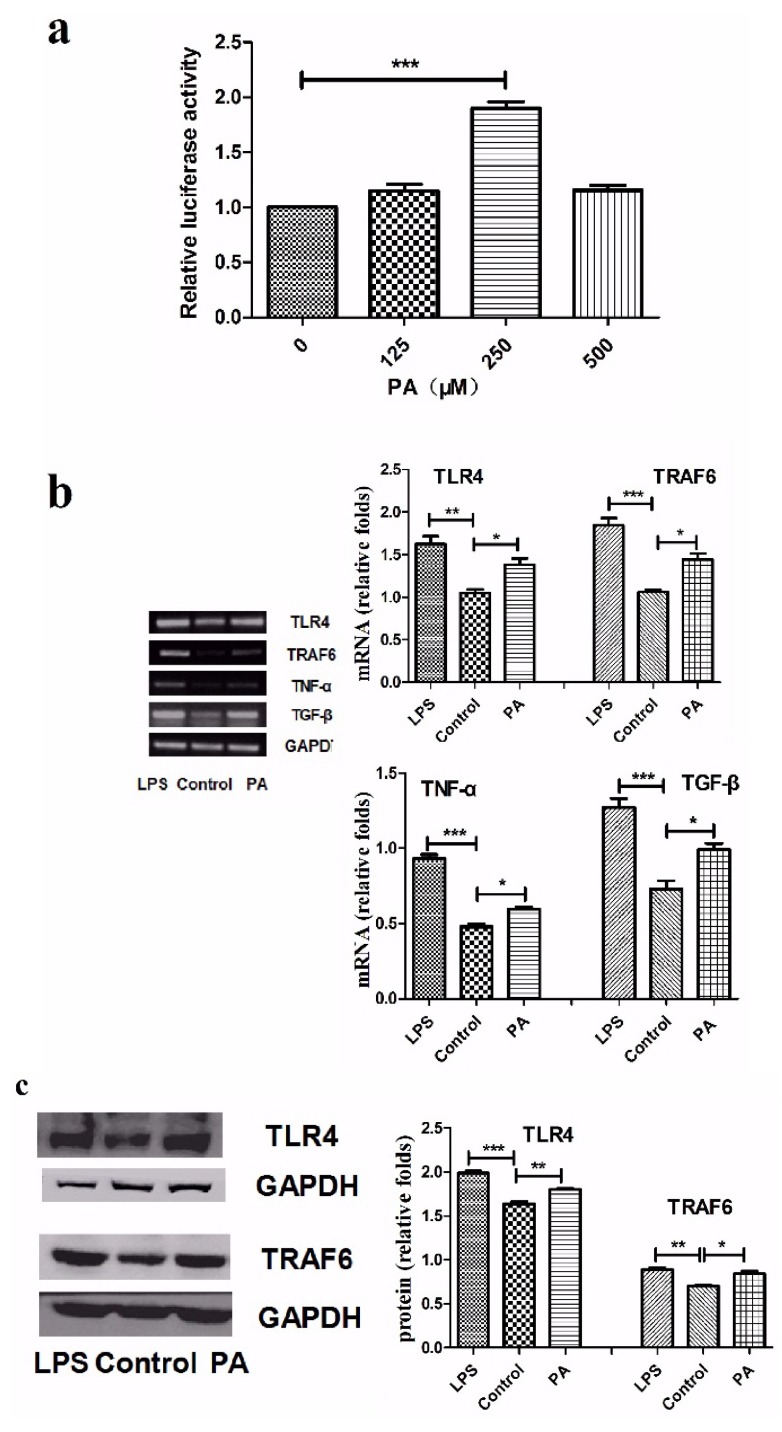

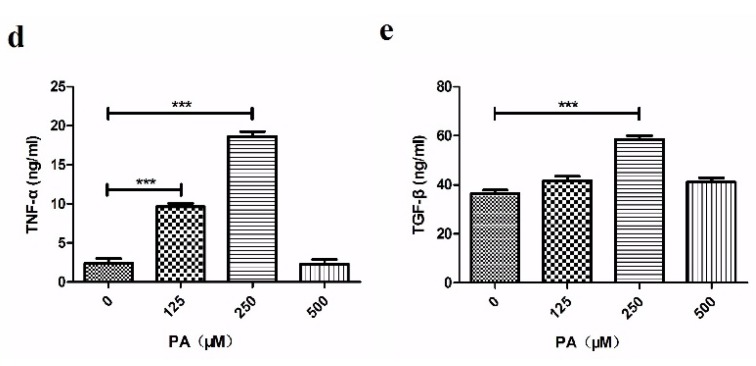
The expression of TLR4, TRAF6, TNF-α and TGF-β in THP-1 after PA exposure. (**a**) THP-1 cells transfected with NF-κB luciferase reporter vector were exposed to different concentrations of PA for 8 h, and the relative luciferase intensity between different groups was detected; (**b**) THP-1 cells were exposed to 250 μM PA for 8 h. Semi-quantitative PCR analysis the mRNA expression of TLR4, TRAF6, TNF-α, TGF-β and LPS is positive control; (**c**) The protein levels of TLR4 and TRAF6 in the THP-1 cells, LPS as a positive control; (**d**,**e**) Supernatants from THP-1 exposed to different concentration PA for 8 h were collected to measure TNF-α and TGF-β by ELISA. Results are mean ± SD. * *p* < 0.05 and ** *p* < 0.01, *** *p* < 0.001.

PA, a saturated free fatty acid (FFA), can also act as a TLR4 ligand to activate TLR4 and induces a series of inflammation-related cascade responses [16]. In our study, we used a NF-κB luciferase report system to explore PA activity of activating TLR pathways. Cell viability was greater than 88% for PA at 125 μM, 250 μM and 500 μM (data not shown).The data showed that luciferase activity significantly evaluated in THP-1 exposing to 250 μM PA for 8 h (Figure 1a). Meanwhile, we detected the mRNA expression of TLR4, TRAF6, TNF-α and TGF-β. TRAF6 is a key signal molecular of TLR4 pathway that can induce nuclear translocation of NF-κB and activator protein (AP)-1, resulting in the production of inflammatory cytokines such as TNF-α. As shown in Figure 1b, mRNA expression of TLR4, TRAF6, TNF-α and TGF-β in PA-stimulated cells were upregulated compared with control. Western blot and ELISA were respectively used to analyze the protein level of these genes. Results also show that TLR4, TRAF6, TNF-α and TGF-β protein expressions were enhanced after exposure to PA (Figure 1c–e). All of these results suggest that PA successfully activates the TLR4 pathway in THP-1 cells.

### 3.2. Palmitic AcidSuppresses miR-194 Expression Which Targets TRAF6

**Figure 2:**
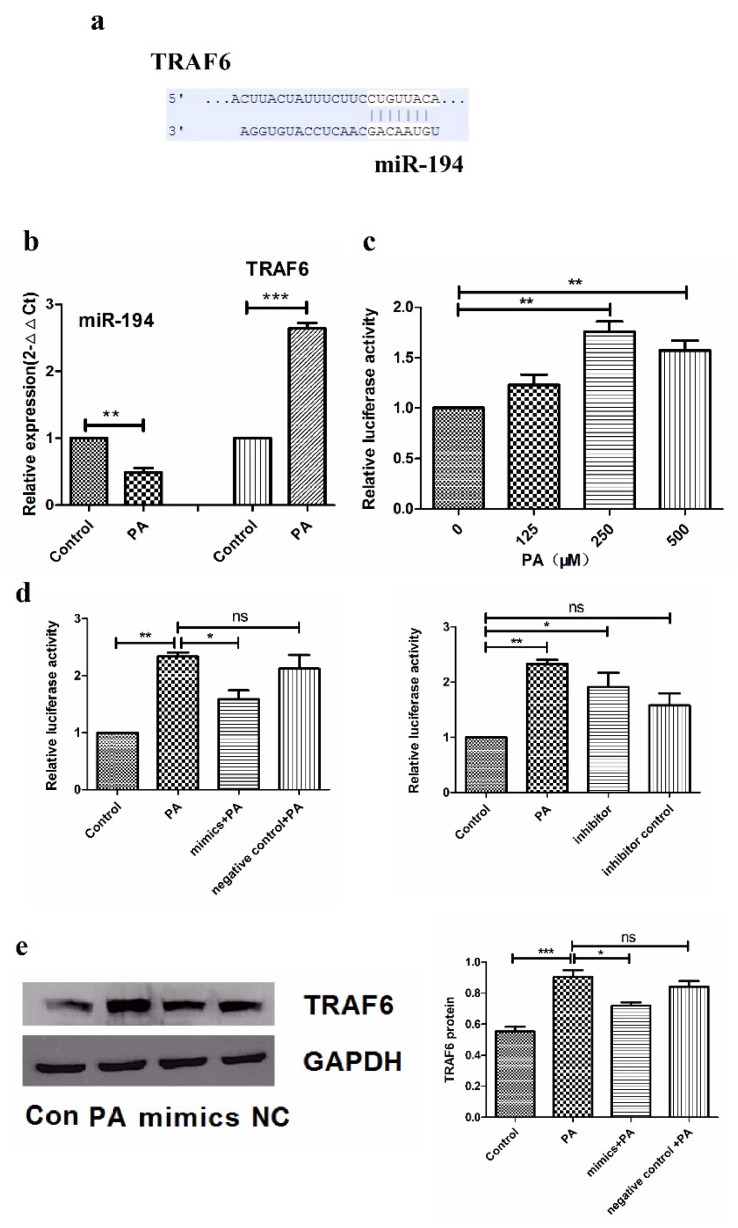

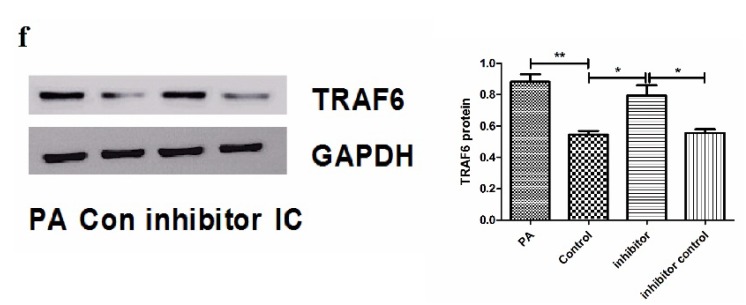
PA induces miR-194 downregulation and miR-194 directly targets TRAF6 in THP-1cells. (**a**) The sequence alignment of miR-194 and its putative target sites of the human TRAF6 mRNA 3′untranslated region were shown; (**b**) THP-1 cells were exposed to 250 μM PA for 8 h and detected the expression of miR-194 and TRAF6 through quantitative real-time PCR; (**c**) Constructed plasmids were transfected into THP-1cells for 24 h and then exposed to different concentration PA for 8 h; (**d**) THP-1 cells were transiently co-transfected with luciferase reporter vectors and miR-194 inhibitor or its control. Constructed luciferase reporter plasmid and miR-194 mimics were co-transfected into THP-1cells for 24 h and then exposed to 250 μM PA for 8 h. All of these whole-cell extracts were prepared for luciferase assay; (**e**,**f**) THP-1 cells were transfected with miR-194 mimics or its control for 24 h and followed 250 μM PA stimulation for 8 h. miR-194 inhibitor and its control were transfected into THP-1 for 32 h. All the above whole-cell extracts were collected for Western blot analysis. Results are mean ± SD. * *p* < 0.05 and ** *p* < 0.01, *** *p* < 0.001.

As common regulators of TLR pathways, microRNAs play an essential role in TLRs homeostasis [17]. In order to investigate miRs which could regulate the TLR4 pathway, we detected some miR expression and found miR-194 downregulation after PA stimulation in THP-1 cells (Figure 2b). Through website (http://www.targetscan.org/) analysis we found TRAF6 is one of the prediction targets of miR-194 (Figure 2a). We used the luciferase report system to identify the direct relationship between miR-194 and TRAF6.

Targets of miR-194 were predicted through software based on seed sequence complementarity. Construct containing 3′UTRs fused behind a luciferase reporter was used to show a direct interaction between miRs and the mRNAs’ target site, and the miRs-mediated repression is measured by changes in activity of 3′UTR containing the luciferase gene in response to co-transfection with miRs [18]. We thus ligated the oligonucleotides which contain TRAF6 mRNA 3′UTR target site into the MulI-XhoI site of pGL3-CM and successfully obtained constructed luciferase reporter pGL3-CM-TRAF6.

The constructed luciferase report plasmid pGL3-CM-TRAF6 was transfected into THP-1, as shown in Figure 2c, and the luciferase expression level significantly evaluated after 250 μM PA treatment. Then miR-194 mimic or inhibitor and their control respectively co-transfected with the plasmid pGL3-CM-TRAF6 into THP-1 cells. According to Figure 2d, miR-194 suppressed luciferase expression which evaluated by PA, meanwhile miR-194 inhibitor enhanced luciferase expression compared with control.

In order to further verify the miR-194 target TRAF6, we transfected miR-194 or miR-194 inhibitor into THP-1 and detected the TRAF6 protein level. As shown in Figure 2e, miR-194 downregulated TRAF6 protein expression, while miR-194 inhibitor upregulated TRAF6 (Figure 2f). All of these results indicated that miR-194 directly target TRAF6.

### 3.3. miR-194 Inhibit the TLR4-Related Cytokine Production

**Figure 3:**
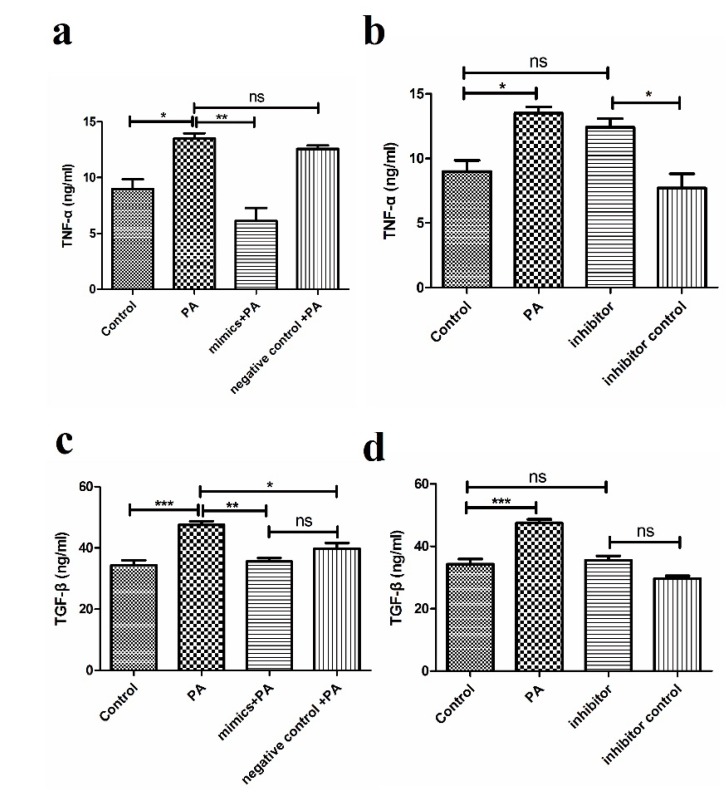
miR-194 reduces TLR4-activated inflammatory cytokine release in THP-1 cells. THP-1 cells were transfected with miR-194 mimics, inhibitor, their control for 24 h and followed 250 μM PA exposure for 8 h. Supernatants were collected to measure TNF-α and TGF-β by ELISA. (**a**,**b**) represent TNF-α; (**c**,**d**) show the change of TGF-β level. Results are mean ± SD. * *p* < 0.05 and ** *p* < 0.01, *** *p* < 0.001.

MiR-194 directly target TRAF6, which could influence downstream cytokine expression such as TNF-α and TGF-β [19]. We transfected miR-194 mimic and its control into THP-1 cells for 24 h followed by PA stimulation, detecting TNF-α and TGF-β levels through ELISA after 8 h. As shown in Figure 3a,c, PA induced TNF-α and TGF-β release, while miR-194 mimic suppressed the cytokine expression. Meanwhile, we also found miR-194 inhibitor enhanced TNF-α expression compared with the negative control but had no obvious effects on TGF-β release (Figure 3b,d). At the same time, there was no significant difference between the normal group and miR-194 inhibitor group in our results. During our experiment, we found miR-194 inhibitor transfection has negative effects on cell growth. That there is no difference between the normal and inhibitor groups maybe correlated with this.

## 4. Discussion

Obesity is always leads tofatty liver disease, insulin resistance and other metabolic diseases. Although simple steatosis is regarded as a benign lesion, inflammatory response induced by free fatty acid is the risk factor of nonalcoholic steatohepatitis (NASH), atherosclerosis and insulin resistance. During the progression of atherosclerosis, the inflammatory mediators promote macrophages transforming into lipid-laden foam cells and drive lesion progression [20]. Moreover, inflammatory cytokines mediate NASH and insulin resistance through hepatic injury and repair, and β cell dysfunction [21]. TLRs recognize a wide range of pathogen-associated molecular patterns including lipids, lipoproteins and proteins [16,22]. Additionally, saturated fatty acid-induced inflammatory responses mainly depend on TLRs. Recent studies demonstrated that the TLR4 pathway in Kupffer cells mediates the progression of simple steatosis to NASH [23]. Followed by TLR4 activation, downstream inflammatory cytokine TNF-α release from KCs subsequently induced hepatic stellate cell (HSC) activation and hepatocyte apoptosis [24,25]. TLR4 activation induced by FFA on HCSs promotes recruitment of KCs and directly augments fibrogenesis [26]. Pathological concentrations of PA always result in necrotic cell death and inflammatory response, which is crucial for the pathogenesis of metabolic syndrome [27,28]. PA activated the TLR4 signal pathway which enhanced ER stress and TGF-β signals and promoted proinflammation cytokine release [29,30]. Our study confirmed that PA, a saturated fatty acid, could induce the activation of the TLR4 signaling pathway in THP-1cells, crucial molecular TRAF6 and cytokine TNF-α upregulation, thereby establishing a cellular model of metabolic inflammation.

Most studies about miR-194 focus on tumor suppression. Recent studies found that miR-194 was downregulated in fibrosis, and more importantly, rescuing miR-194 decreased stellate cell activation through targeting rac1 [31]. Rac1 is a member of the Rho family of small GTP-binding proteins, which is required for HSC proliferation and activation [31]. In our study, miR-194 downregulated by PA and rescuing miR-194 decreased TNF-α expression through targeting TRAF6. TRAF6 is a downstream signal molecular of TLR4, and phosphorylation of TRAF6 results in the nuclear translocation of the transcriptionfactor NF-κB which results in proinflammatory cytokines such as TNF-α production [19]. Meanwhile, miR-194 inhibitor upregulated TRAF6 expression and promoted TNF-α expression. Taken together, our results indicate that miR-194 attenuates TLR4 signaling pathway via targeting TRAF6 during PA stimulation in THP-1 cells. We identifymiR-194 as having a new role that involves inflammation induced by PA.

Recent studies indicated that TLR4 enhanced TGF-β signaling and hepatic fibrosis [29]. Kupffer cells are a main source of TGF-β in the liver, which promotes HSC activation and fibrogenesis [32]. Moreover, another study found that LPS caused a potent decrease in miR-29 expression in normal fibroblasts that were TLR4 dependent, and rescuing miR-29 in LPS-treated fibroblasts abrogated the stimulation of collagen gene expression in the presence of TGF-β, directly implicating repression of miR-29 as a contributor to exaggerated response to TGF-β [33]. As previously studied, in accompaniment with TLR4 activation, TGF-β expression was upregulated in THP-1 cells during PA stimulation in our study. MiR-194 decreased TNF-α expression through targeting TRAF6 but had no effect on TGF-β expression. It suggests that the regulation of miR-194 in TLR4 is limited and interactions of the intracellular signaling pathways are complex.

Taken together, based on the PA-activated TLR4 pathway, we found miR-194 inhibited the TLR4 pathway through targeting a key signal molecule TRAF6. As a TLR4 common downstream cytokine, TNF-α was decreased by miR-194. Our results showed that miR-194 attenuated PA-induced cytokine TNF-α release through suppressing a key molecule TRAF6 in the TLR4 pathway, and both miR-194 and TRAF6 are thus potential targets in the developing therapy strategy for PA-related disease.

## 5. Conclusions

Our study demonstrates that PA activates the TLR4 signal pathway, upregulates a key molecule TRAF6 and cytokines TNF-α and TGF-β, and also downregulates miR-194 expression in THP-1 monocytic cells. Overexpression of TNF-α and TGF-β results in cell injury, accumulation of immune cells, inducing more proinflammatory cytokines and production of fibrosis-related proteins, which promote obesity-related disease development. We also found that TRAF6 was a target gene for miR-194 which attenuates PA-induced TRAF6 upregulation and cytokine TNF-α release. These results suggest that after PA stimulation, downregulated miR-194 results in TRAF6 (a key molecule in the TLR4 pathway) overexpression and further mediates downstream cytokine expression. Both miR-194 and TRAF6 are therefore potential targets that can contribute to the therapy strategy of PA-related diseases.
